# Comparison of Two Analytical Platforms for CSF Biomarkers of Alzheimer's Disease

**DOI:** 10.1155/2014/765130

**Published:** 2014-05-29

**Authors:** Jose Antonio Monge-Argilés, Carlos Muñoz-Ruiz, José Sánchez-Payá, Ruth Gasparini Berenguer, Maria Empar Blanco Cantó, Carlos Leiva-Santana

**Affiliations:** ^1^Department of Neurology, University General Hospital of Alicante, Avenida Pintor Baeza 12, 8^a^ C, 03010 Alicante, Spain; ^2^Laboratory of Immunology, University General Hospital of Alicante, Avenida Pintor Baeza 12, 8^a^ C, 03010 Alicante, Spain; ^3^Preventive Medicine Service, University General Hospital of Alicante, Avenida Pintor Baeza 12, 8^a^ C, 03010 Alicante, Spain

## Abstract

Cerebrospinal fluid (CSF) biomarkers of Alzheimer's disease (AD) are currently being assessed with two different assays. Our objective was to study if there is a correlation between values obtained by both techniques, to compare their validity and search for conversion factor between values obtained for every protein. We compared the performances of two commonly used platforms, an enzyme-linked immunosorbent assay (ELISA) and a multiplex (xMAP) technology for measurement of CSF A*β*_1–42_, total tau (T-tau), and phosphorylated tau 181 (P-tau_181p_) proteins, in 30 AD patients and 28 control subjects. The relations between the variables of both techniques were evaluated using the Spearman *p* correlation coefficient (*α* = 0.05). Receiver operating characteristic and area under the curve (AUC) analyses were calculated for the variables of both techniques. The two assays platforms yielded different absolute values for the various analytes, always higher in ELISA. We found some correction factor between values: 2,1- to 3-fold for A*β*_1–42_; 4,1- to 4,6-fold for T-tau; and 1,4- to 1,6-fold for P-tau_181p_. In addition, those values were highly correlated (A*β*_1–42_: *r* = 0.70, *P* < 0.01; T-tau: *r* = 0.90, *P* < 0.01; P-tau_181p_: *r* = 0.85, *P* < 0.01) and the AUC for the variables showed very similar values. In conclusion, the results obtained with ELISA and xMAP platforms were highly correlated and its validity is very similar. Differences in absolute values point to the need for a clear description of the technique used. Moreover, we found some conversion factor between values of every protein that may be useful for transformation between both techniques.

## 1. Introduction

Accurate and early differentiation of Alzheimer's disease (AD) from other dementia illnesses, psychiatric disorders, or even mild cognitive impairments (MCI) is becoming increasingly important [1] because symptomatic drugs are specifically available for AD patients and disease modifying treatments, based on altered amyloid metabolism, are being developed [2].

Cerebrospinal fluid (CSF) levels of A*β*_1–42_ protein (A*β*), total tau protein (T-tau), and phospho-tau protein 181p (P-tau) have been shown to have a diagnostic utility for discriminating AD dementia cases from cognitively normal controls [3], early recognition of patients with MCI due to AD or dementia due to AD [4, 5].

These CSF biomarkers of AD are currently being assessed with two different platforms: enzyme-linked immunosorbent assay (ELISA) and a multiplex technology (xMAP). Nevertheless, there are differences in absolute values between both techniques and no conversion factor exists to recalculate results obtained with one of them to those from the other [3, 6].

Our aim was to study if there is a correlation between values obtained by both techniques, to compare their validity and search for conversion factors between them.

## 2. Patients and Methods

### 2.1. Study Design

The study is a cross-sectional study.

### 2.2. Patients

Patients were divided as follows:

- thirty probable NINCDS-ADRDA AD patients [7] in out-patients clinic of the University General Hospital of Alicante (Spain), referred by a general practitioner or a neurologist;
- twenty-eight control subjects who were to undergo spinal anaesthesia for traumatological or urological nonmalignant conditions, without cognitive deterioration.

They all underwent physical and neurological examination, neuropsychological studies, assessment of depression using the Yesavage scale, blood test, and LP. Cerebral magnetic resonance imaging (MRI) was made only in AD patients.

We included subjects over the age of fifty-five. In the control group, no patient had subjective memory loss, all minimental state examination (MMSE) test results were above twenty-seven, and the informant questionnaire on cognitive decline in the elderly (IQCODE) was under 78 [7]. The neuropsychological criteria for probable AD were a MMSE under 22 and IQCODE over 84. Before inclusion, informed consent was obtained for LP and for examination of CSF samples for research purposes.

The exclusion criteria were dementia from other etiologies, anticoagulant therapy or failure to obtain informed consent, and a score greater than five using the Yesavage scale of depression.

### 2.3. Procedures

The neurologist responsible for each patient made a diagnosis of probable AD after clinical and neurological examination, blood test, MRI, and neuropsychological examination. This one included MMSE test, the IQCODE, Rey auditory verbal learning, California verbal learning, trail making test, and the geriatric depression scale of Yesavage. With these tests, the evaluation of memory, language, executive function, attention, and visuoconstructive capacity was made. Alteration of one function was defined as a *Z* result of −1.5 or less, which was at least 1.5 standard deviations below the mean of the control subjects, in at least one of the tests used to evaluate that function. The neuropsychological tests done in control group were the same as in patients group.

### 2.4. Extraction and Analysis of CSF

The extraction of CSF was performed between February 2008 and February 2010.

In AD patients, LP was performed with a 20 × 3.5 gauge needle in out-patient clinic, between 9 a.m. and 2 p.m. They were premedicated with 5 mgr of oral diazepam and lumbar skin anaesthesia.

In control subjects, the CSF (±1 mL) was obtained in the operating theatre by the anaesthetist performing spinal anaesthesia, between 9 a.m. and 2 p.m. They were premedicated with intravenous diacepam and metamizole.

The CSF sample was collected in standard tubes and centrifuged before being aliquoted in polypropylene tubes and frozen at −80°C. Obvious sanguinolent CSF was discarded. Samples were frozen within an hour of the procedure.

The analysis of CSF samples was made at the end of the collection, in doublets, for every technique.

### 2.5. Quantification of Variables

The two analytical platforms used were ELISA (INNOTEST) and xMAP (INNOBIA Alzbio3) from INNOGENETICS (Ghent, Belgium). The quantification of variables (A*β*, T-tau, and P-tau) for the two analytical platforms was made from the same tube. The details of reagent combination in xMAP have been previously published [8].

### 2.6. Statistical Analysis

The Kolmogorov-Smirnov test was used to analyse the distribution of each variable. After that we analyzed

- Spearman *p* correlation coefficient (*α* = 0.05);
- receiver operating characteristic (ROC) curve analysis which was calculated for the variables of both techniques.

In all hypotheses, a *P* value of less than 0.05 determined statistical significance. The statistical package SPSS version 19.0 was employed.

## 3. Results

Thirty probable AD patients and 28 control subjects were included. No statistical differences were seen in the age of both groups. There were twenty percent more females in AD group than in control group, as shown in Table 1.

In Table 2, we show the results of each biomarker in the two analytical platforms. We observed that A*β* concentrations were significantly decreased and T-tau and P-tau concentrations were significantly increased in AD patients, in comparison with control subjects, both in the ELISA and in the xMAP assays.

When we compared the results between both platforms (Table 3) we found that ELISA A*β* protein concentration is about 2,1-fold more than xMAP for AD patients and is about 3-fold more for control subjects. Moreover, ELISA T-tau concentration is about 4,6-fold more than xMAP for AD patients and 4,1-fold for control subjects. Finally, ELISA P-tau concentration is about 1,4-fold more than xMAP for AD patients and 1,6 for control subjects.

The Spearman *p* correlation coefficient between both platforms was 0.70 for A*β* protein, 0.90 for T-tau, and 0.85 for P-tau, with a significant level less than 0.01 in every correlation (Table 4).

**Figure 1:**
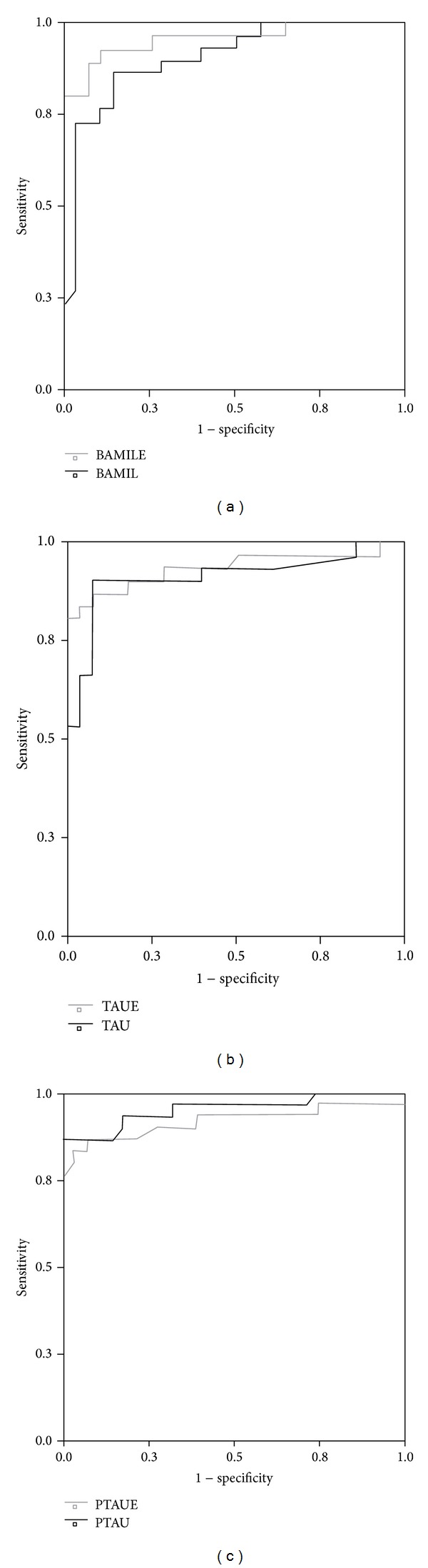
ROC curve analysis. Comparison of area under the curves (AUCs) between ELISA (BAMILE, TAUE, PTAUE) and xMAP (BAMIL, TAU, PTAU) analysis results. No significant differences between AUCs (*P* < 0.05). Sensitivity % (at 80% specificity): ELISA: A*β*_1–42_ = 90; Tau protein = 85; P-tau protein = 84 xMAP: A*β*_1–42_ = 82; Tau protein = 83; P-tau protein = 85.

Finally, when we compared the area under the curve (AUC), in the ROC curve analysis for every biomarker, we did not find statistical differences between both platforms (Figure 1).

## 4. Discussion

In this study, we compared the performances of two currently used commercial assays for CSF biomarkers of AD. We concluded that they performed almost equally well in discriminating control subjects from AD patients. We did not find statistical differences between AUCs for every protein of both platforms. These results are in accord with those published recently [3, 6, 9, 10]. Due to the technological differences, because in xMAP is simultaneous, and in ELISAs are individualized measures of the three proteins, we have not a preference between them and we must leave the choice of the technique to every laboratory, depending on its availabilities.

Correlations between the two assay types were very good for T-tau and P-tau (0.90 and 0.85, resp.) and moderate for A*β* (0.70). These results are very similar to other previous studies [3, 10]. The explanations for those findings comprise the use of different combinations of antibodies in the respective assays, the different interaction with antigen of antibodies coated on a microsphere (xMAP) versus antibodies coated on microtiter plate, and differences in assay procedures.

As described before, the two methods produced different absolute values for the various biomarkers (approximately 2,1- to 4,6-fold), always higher in ELISA than in xMAP technology. The reasons of this variability may be the same exposed in the previous paragraph. A recent study [6] suggested that a constant correction factor could be used to convert results obtained with the xMAP assays to ELISA values, but some other studies [3] did not reach the same results. Despite the fact that we did not find a constant correction factor for the three proteins, we described a near similarity between correction factors, always in the same unit, for every biomarker. These results are very similar to those obtained in other two similar studies recently published [9, 10].

In agreement with multiple previous studies [1, 4, 5, 8], we observed that A*β* concentrations were significantly decreased, and T-tau and P-tau concentrations were significantly increased in AD patients, in front of control subjects, both in the ELISA and in the xMAP assays. However, there is an overlap between AD and control subjects. It may be explained because all control subjects theoretically can have preclinical AD [4]. As limitations of this study, we have to include that our control group has not been examined with neuroimaging tests, to exclude possible future cognitive impairment.

## 5. Conclusions

The results obtained with ELISA and xMAP were highly correlated and their validity was very similar. However, differences in absolute values point to the need for a clear description of the technique used. Despite this, we find some possible conversion factors for every CSF biomarker but we need confirmation.

## Figures and Tables

**Table 1:** Study participant demographics.

Characteristics	AD patients	Control subjects	Signification level
Participants number	30	28	—
Mean (SD) age at LP, years	72.66 (6.83)	70 (7.43)	N.s.
Female, %	63	40	0.01
Medical conditions:			
HTA	8	7	
DM	10	10	
HPL	8	10	
Hip replacement	0	12	
Knee replacement	0	10	
Prostatic adenoma	0	6	
Mean MMSE Folstein ± SD	23 ± 1.2	28 ± 0.5	0.01
Mean IQCODE ± SD	82 ± 5	68 ± 3	0.01
Mean Yesavage depression scale ± SD	2.1 ± 0.5	2.3 ± 0.7	N.s.

**Table 2:** CSF biomarker concentrations.

Proteins	INNOTEST		INNOBIA	
	AD patients	Control subjects	AD patients	Control subjects
A*β*_1–42_	645.5 ± 282.83	1659.6 ± 660.17	297.7 ± 99.8	510.88 ± 125.75
T-tau	572.73 ± 438.61	167.23 ± 46.5	122.0 ± 86.64	40.76 ± 15.75
P-tau_181p_	86.71 ± 33.57	41.03 ± 10.45	61.0 ± 23.11	25.0 ± 4.44

**Table 3:** Folds in INNOTEST more than in INNOBIA Alz-bio3.

CSF proteins	AD patients	Control subjects
A*β*_1–42_	2.1	3
T-tau	4.6	4.1
P-tau_181p_	1.4	1.6

**Table 4:** Correlation between INNOTEST and INNOBIA Alz Bio-3: AD patients and control subjects.

CSF proteins	*R*	Signification level
A*β*_1–42_	0.70	*P* < 0.01
T-tau	0.90	*P* < 0.01
P-tau	0.85	*P* < 0.01

Spearman *p* correlation coefficient (*α* = 0.05).

## Abbreviations

A*β*: A*β*_1–42_ protein
T-tau: Total tau protein
P-tau: Phospho-tau protein 181p.
